# Supplementary material for: Influence of Hydrogen‐Incorporation on the Bulk Electronic Structure and Chemical Bonding in Palladium
Source: Adv Sci (Weinh). 2026 Feb 10;13(20):e22098. doi: 10.1002/advs.202522098 (PMC13067790; doi:10.1002/advs.202522098)
Supplement: Supplementary file 1 — Supporting File: advs74182‐sup‐0001‐SuppMat.pdf. [file ADVS-13-e22098-s001.pdf]

Influence of Hydrogen-Incorporation on the Bulk Electronic Structure and Chemical Bonding in Palladium
Supplementary Information

L. J. Bannenberg,[1] F. Garcia-Martinez,[2] P. Lömker,[3,4,2] R. Y. Engel,[3,4]
C. Schlueter,[2] H. Schreuders,[1] A. Navarathna,[1] L. E. Ratcliff,[5,6] and A. Regoutz[7,8,*]

[1]*Faculty of Applied Sciences, Delft University of Technology, Mekelweg 15, 2629 JB Delft, The Netherlands.*
[2]*Photon Science, Deutsches Elektronen-Synchrotron DESY, 22607 Hamburg, Germany.*
[3]*Department of Physics, Stockholm University, 10691 Stockholm, Sweden.*
[4]*Wallenberg Initiative Materials Science for Sustainability, Department of Physics, Stockholm University, 114 28 Stockholm, Sweden.*
[5]*Centre for Computational Chemistry, School of Chemistry, University of Bristol, Bristol BS8 1TS, United Kingdom.*
[6]*Hylleraas Centre for Quantum Molecular Sciences, Department of Chemistry, UiT The Arctic University of Norway, N-9037 Tromsø, Norway.*
[7]*Department of Chemistry, University of Oxford, Inorganic Chemistry Laboratory, Oxford OX1 3QR, United Kingdom.*
[8]*Department of Chemistry, University College London, London WC1H 0AJ, United Kingdom.*

I. STRUCTURAL CHARACTERISATION

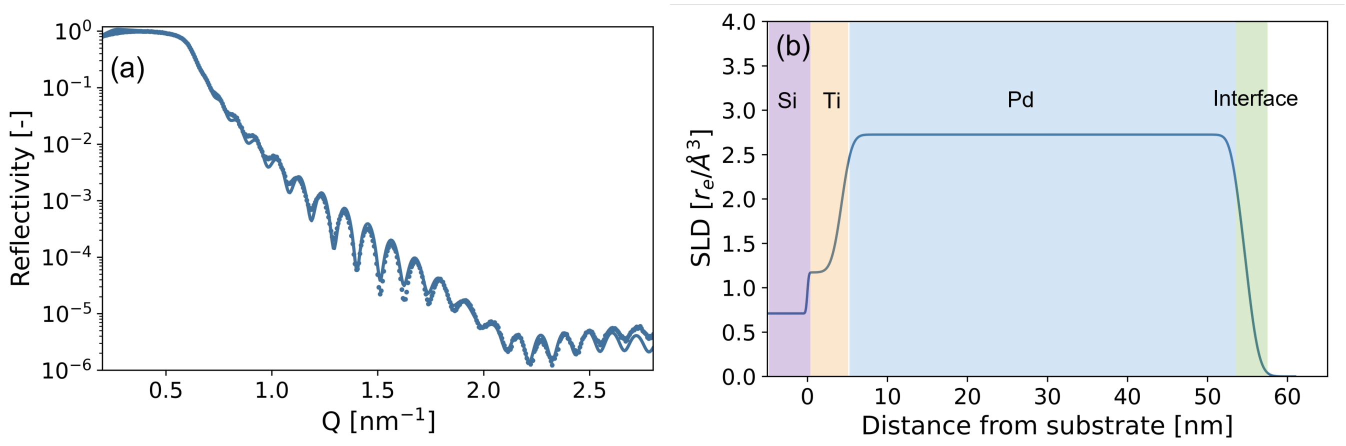

FIG. 1: X-ray reflectometry (XRR) results of the sample composed of a Ti and Pd layer. (a) X-ray reflectometry data as a function of the momentum transfer Q. The points indicate the measured data, while the continuous line represents a fit to the data. (b) Corresponding scattering length density profile (SLD) as a function of the distance from the substrate and obtained from fitting the experimental data of (a) to a model. The SLD is, in the case of X-rays, roughly proportional to the mass density of the film.

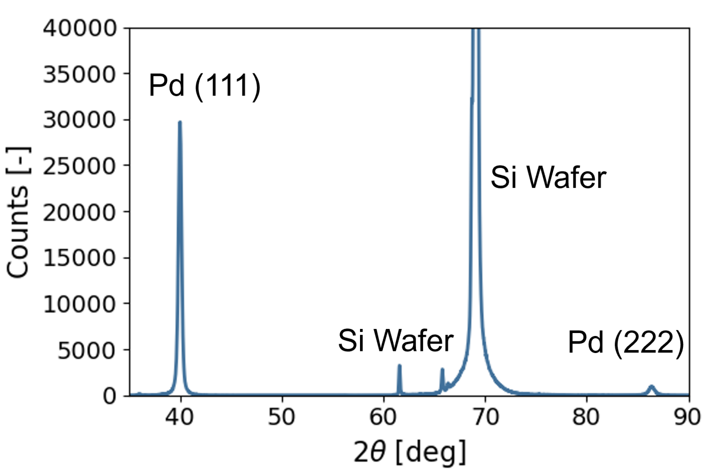

FIG. 2: Ex-situ X-ray diffraction measurements of the sample composed of a 4 nm Ti and a 50 nm Pd layer on a Si wafer.

II. AP-HAXPES & THEORY

TABLE I: One-electron corrected photoionisation cross sections, σ, and number of electrons, n(e⁻), of Pd s, Pd p, Pd d, and H s valence states. Values from Scofield from Ref. XX are interpolated from 4 and 5 keV listed values for the experimental photon energy of 4.596 keV, with the lowest Pd s and p orbitals being the deeper, occupied 4s and 4p states. The "Scofield In correction" approach is similar to that implemented in a previous paper on W metal, XX where the unoccupied 5s and 5p cross sections are estimated extrapolating from the respective orbital cross sections for In.

**Scofield**	Pd 4s	Pd 4$p_{1/2}$	Pd 4$p_{3/2}$	Pd 4$d_{3/2}$	Pd 4$d_{5/2}$	H s
σ (4 keV)	647	526	479	81	75	0
σ (5 keV)	415	304	273	38	35	0
σ (4.596 keV)	553.35	436.16	395.62	63.75	58.77	0.04
n(e⁻)	2	2	4	4	6	2
**Scofield In correction**	In 5s	In 5$p_{1/2}$	In 5$p_{3/2}$	Pd 4$d_{3/2}$	Pd 4$d_{5/2}$	H s
σ (4 keV)	81	37	30	81	75	0
σ (5 keV)	53	22	18	38	35	0
σ (4.596 keV)	69.29	30.84	25.26	63.75	58.77	0.04
n(e⁻)	2	0.33	0.67	4	6	2

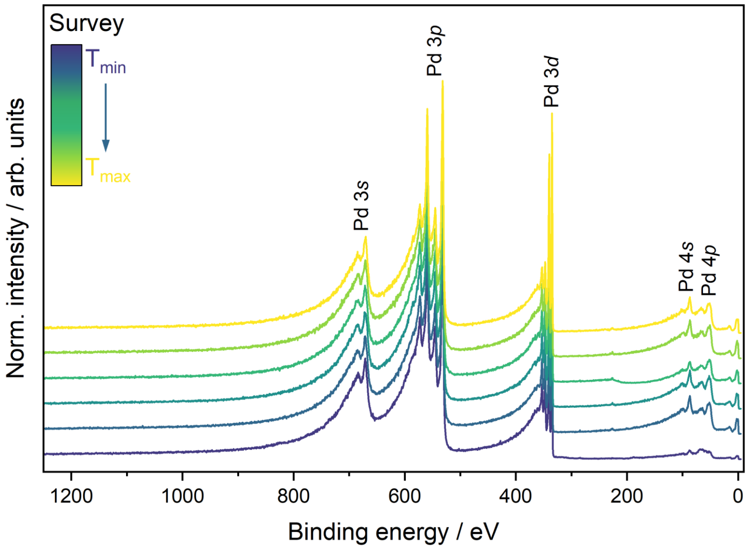

FIG. 3: AP-HAXPES survey spectra collected after exposure to 200 mbar H_2, cooling to room temperature, followed by heating. All major core levels are indicated. The spectra are normalised (0,1) and stacked.

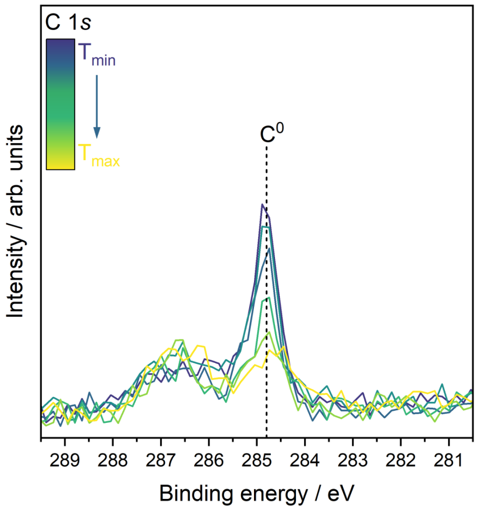

FIG. 4: AP-HAXPES C 1s spectra collected after exposure to 200 mbar H_2, cooling to room temperature, followed by heating.

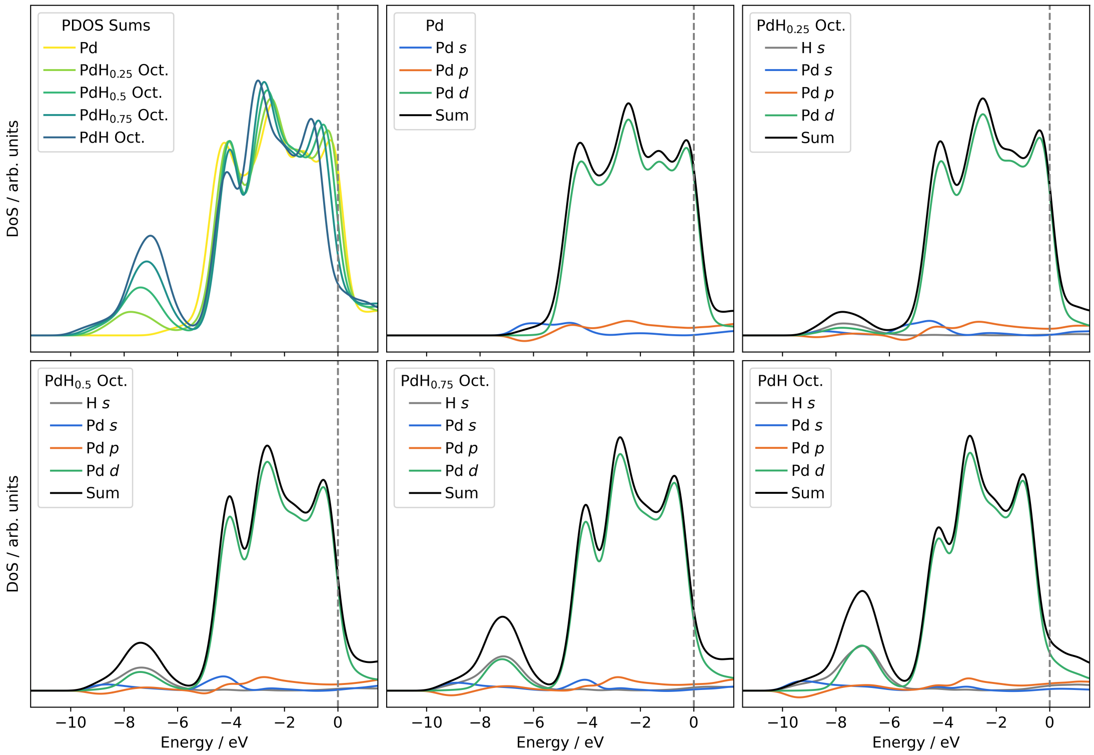

FIG. 5: Projected density of states (PDOS) for the different H concentrations, with H occupying the octahedral sites. The upper left plot shows a comparison of the sum of the PDOS for all systems, while the remaining plots show the PDOS for individual systems. The PDOS have been aligned to the calculated Fermi energy, which is indicated with a dashed vertical line.

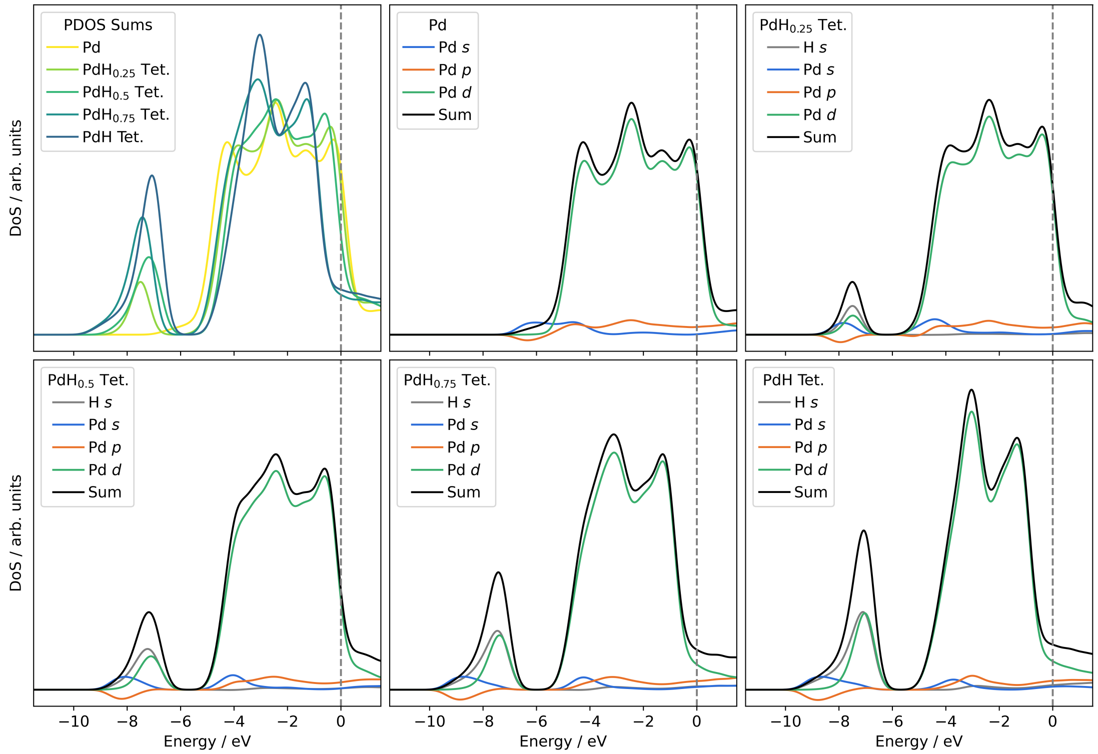

FIG. 6: Projected density of states (PDOS) for the different H concentrations, with H occupying the tetrahedral sites. The upper left plot shows a comparison of the sum of the PDOS for all systems, while the remaining plots show the PDOS for individual systems. The PDOS have been aligned to the calculated Fermi energy, which is indicated with a dashed vertical line.

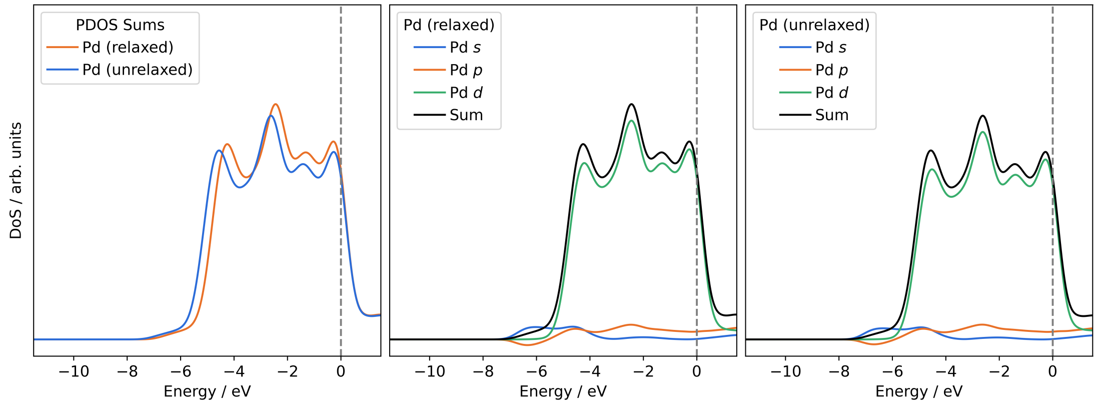

FIG. 7: Projected density of states (PDOS) for Pd with both its experimental ('unrelaxed') and DFT-relaxed lattice parameter. The left plots show a comparison of the sum of the PDOS for both systems, while the remaining plots show the PDOS for individual systems. The PDOS have been aligned to the calculated Fermi energy, which is indicated with a dashed vertical line.

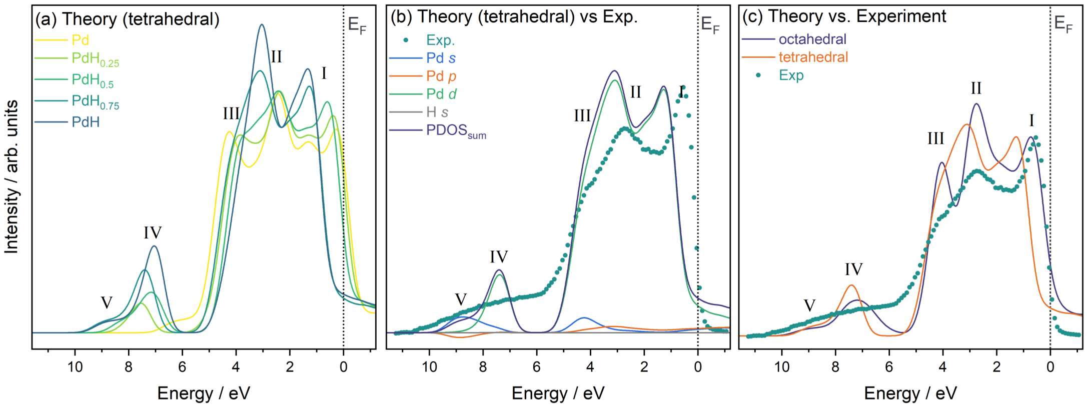

FIG. 8: Comparison of PDOS for hydrogen occupying octahedral or tetrahedral sites, including (a) photoionisation cross-section corrected sums of theoretical PDOS for the Pd to PdH series assuming tetrahedral occupation, (b) comparison of the calculated PDOS for $PdH_{0.75}$ and the AP-HAXPES VB spectrum with the highest hydrogen loading, and (c) a comparison of the same experimental dataset with PDOS assuming either octahedral or tetrahedral occupation. All PDOS are corrected using the Scofield In σ correction. The position of the Fermi energy E_F at 0 eV is shown in all subfigures and Roman numerals are used to indicate the main spectral features observed. In (c) PDOS and VB spectrum are normalised to the height of feature I.

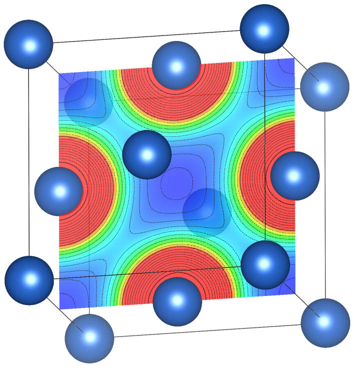

(a) Pd

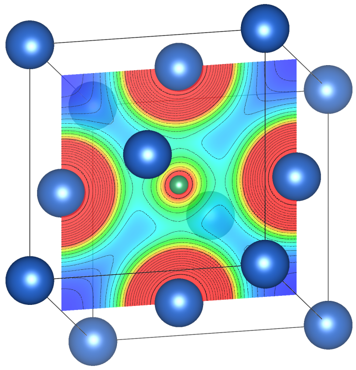

(b) $PdH_{0.25}$

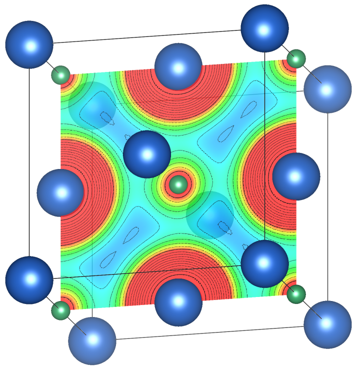

(c) $PdH_{0.5}$

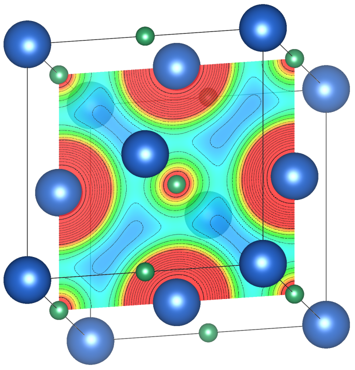

(d) $PdH_{0.75}$

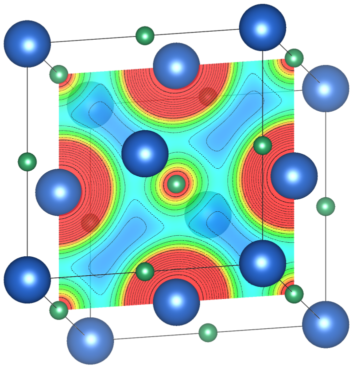

(e) PdH

FIG. 9: Depiction of the calculated electronic densities for (a) Pd and (b)-(e) PdH_x for different x, with H occupying the octahedral positions. Pd (H) atoms are depicted in blue (green).

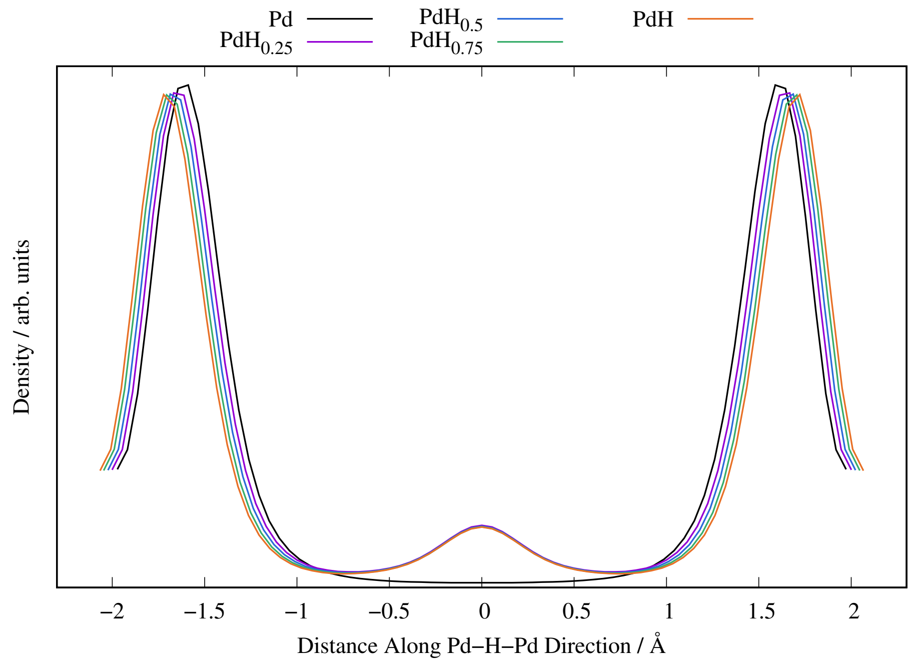

FIG. 10: Plot of the calculated electronic densities along the Pd-H-Pd direction, i.e. (0.0,0.5,0.5) to (1.0,0.5,0.5), for Pd and PdH_x for different x, with H occupying the octahedral positions. For ease of reading, the x axis origin is at (0.5,0.5,0.5), i.e. centred around the H position where a H atom exists.

TABLE II: DFT-calculated Mulliken and Bader charges for the structures where H occupies the octahedral positions. Values are given for each of the four Pd atoms in the unit cell since these sometimes differ between Pd atoms, while the charges are the same across all H atoms in each case.

	Pd_1	Pd_2	Pd_3	Pd_4	H
**Mulliken**					
Pd	0.000	0.000	0.000	0.000	-
$PdH_{0.25}$	0.103	-0.007	-0.007	-0.007	-0.082
$PdH_{0.5}$	0.114	0.114	-0.042	-0.042	-0.072
$PdH_{0.75}$	-0.098	0.097	0.097	0.097	-0.064
PdH	0.061	0.061	0.061	0.061	-0.061
**Bader**					
Pd	0.000	0.000	0.000	0.000	-
$PdH_{0.25}$	0.108	-0.012	-0.012	-0.012	-0.073
$PdH_{0.5}$	0.078	0.078	-0.013	-0.013	-0.065
$PdH_{0.75}$	0.111	0.027	0.027	0.027	-0.064
PdH	0.073	0.073	0.073	0.073	-0.073

* anna.regoutz@chem.ox.ac.uk
